# Ambiguous Role of p53 in Transcription-Dependent Tumor Cell Death

**DOI:** 10.3390/ijms27020769

**Published:** 2026-01-12

**Authors:** Angelina A. Romanova, Tatyana A. Grigoreva, Anastasia D. Zenina, Anna D. Smirnaya, Kira Y. Margolina, Aleksandra Sagaidak, Vyacheslav G. Tribulovich

**Affiliations:** Laboratory of Molecular Pharmacology, St. Petersburg State Institute of Technology (Technical University), St. Petersburg 190013, Russia

**Keywords:** p53, cell death, apoptosis, necrosis, autophagy

## Abstract

Currently, research in anti-cancer therapy remains a priority. This is driven by two main challenges: the difficulty of modeling and developing targeted or precision drugs and the multiple, often unpredictable, body responses to treatment. The primary objective of modern anti-cancer drugs is the induction of cancer cell death. One of the key regulators of cell death is the tumor suppressor protein p53. This protein is a well-known transcription factor encoded by *TP53*. Despite the fact that p53 is generally considered a pro-apoptotic inducer, it also regulates cell death pathways such as necrosis and autophagy. Given the diversity of p53-mediated cell death pathways, establishing a specific activated mechanism is a necessary step in developing effective anti-cancer drugs, since certain types of cell death can cause adverse outcomes in patients, including infection, sepsis, tumor progression and metastasis. The review summarizes knowledge about p53-dependent cell death mechanisms and the p53 transcriptional targets that are involved. It also describes shared molecular pathways among apoptosis, necrosis, and autophagy, as well as the methods and markers used to distinguish one type of cell death from another.

## 1. Introduction

Known as the “guardian of the genome”, p53 is a widely studied protein responsible for maintaining genomic stability through the suppression of various pathological processes, including carcinogenesis. In response to cellular stress, p53 maintains homeostasis by affecting the cell cycle, senescence, and other processes through direct interaction with proteins or transcriptional activation of target genes. When the level of damage exceeds the physiologically acceptable level, p53 triggers the mechanisms of cell death [1].

p53 primarily functions as a transcription factor by binding to canonical DNA sequences in the promoters of its target genes, known as p53 response elements (REs) (RRRCWWGYYY: R = G/A; W = A/T; Y = C/T). In addition to its role as a transcription factor, p53 can induce cell death directly through protein–protein interactions. This mechanism facilitates an ‘emergency’ cellular response to critical damage, bypassing the time-consuming, multi-step processes of transcription and translation. Although transcription-independent pathways of p53 contribute significantly to cell fate determination, this review aims to provide a detailed examination of its transcriptional functions. The cell fate is determined by the nature of the stress stimulus, as well as by the post-translational modifications, quantity, and cellular localization of p53 [2,3]. Moreover, p53 can simultaneously or sequentially initiate several cell death mechanisms [4].

**Figure 1:**
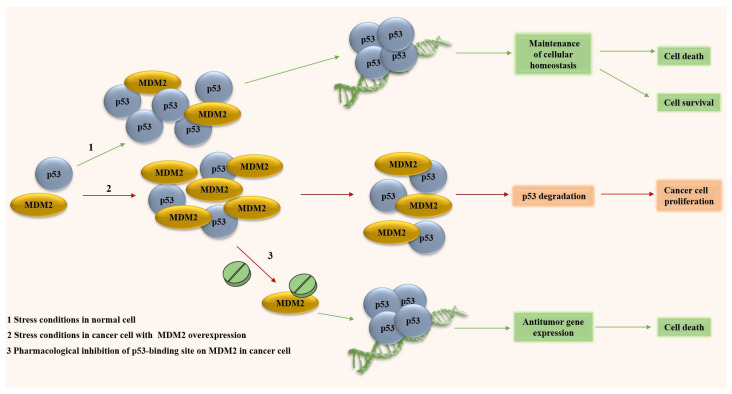
The p53-MDM2 interaction in cancer. p53—transcription factor; MDM2—E3-ubiquitin ligase. Green arrow: anti-tumor mechanism; red arrow: pro-tumor mechanism.

Many malignant tumors exhibit impaired anti-cancer mechanisms, caused by both mutations in the *TP53* and the suppression of its protein activity. In 30–50% of cancer cases, p53 activity is suppressed due to the amplification of its antagonist, the E3-ubiquitin ligase MDM2 [3]. MDM2 directs p53 to proteasomal degradation via polyubiquitination and to export from the nucleus via monoubiquitination [5]. The overexpression of *MDM2* in both scenarios results in the excessive suppression of transcriptional functions of p53, leading to abnormal cell growth and malignant transformation (Figure 1).

Reactivation of the transcriptional activity of p53 represents a promising therapeutic strategy for the treatment of hyperproliferative diseases. The pharmacological stabilization and accumulation of p53 is accomplished through the disruption of its interaction with MDM2. The function of p53 can be restored by small-molecule compounds that either competitively bind to the p53-binding domain of MDM2 (e.g., Nutlins, SP-141) or directly to p53 (e.g., RITA) [6,7]. In the latter case, it is also possible to restore the functions of mutant p53 [8]. Therefore, artificially inhibiting the p53-MDM2 interaction represents a promising strategy for targeting cancers with *MDM2* overexpression, and in some cases, those with mutant forms of p53. Irrespective of the implemented approach, the ultimate goal of any such therapy is the p53-mediated death of cancer cells.

## 2. Types of Cell Death and the Role of p53 in Mediating Them

To date, there are a number of independent classifications of cell death, which are based on the following [9,10]:

- Various morphological and structural characteristics,
- The involvement of caspases,
- Process controllability,
- Molecular pathways leading to cell death.

This variety of classifications is justified by the complex nature of cell death and the vagueness of the criteria used to define it.

**Figure 2:**
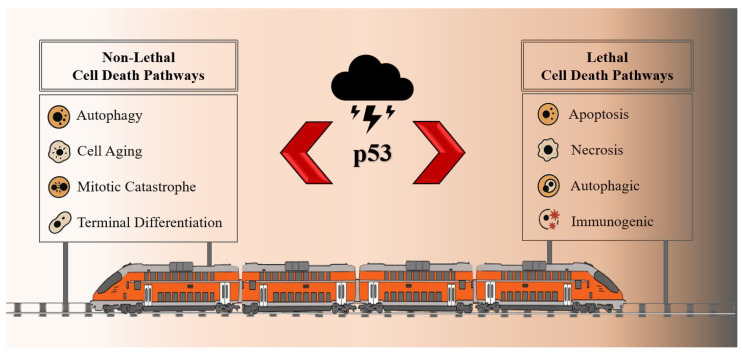
Classification of cell death in which p53 is directly or indirectly involved, including switching between pro-survival and pro-death processes. p53—transcription factor.

The Nomenclature Committee on Cell Death (NCCD) regularly updates recommendations on the classification of cell death. According to them, two groups can be distinguished: lethal (leading to cell death) and non-lethal. (Figure 2). The lethal group includes apoptosis, necrosis (including necroprotosis, ferroptosis, pyroptosis, and parthanatosis), entosis, netosis, autophagic (lysosomal) and immunogenic cell death. The non–lethal group—autophagy, cellular aging, mitotic catastrophe, and terminal differentiation [10].

Identifying a specific type of cell death is a complex task, since different types may involve similar metabolic pathways, which can lead to the same ultimate fate—cell death. Based on morphological features in physiological and pathological processes, three types of cell death have been distinguished: apoptosis, necrosis (including necroptosis, ferroptosis, and pyroptosis), and autophagic cell death [11]. These types have fundamental differences not only in terms of morphology, but also in terms of the possible consequences for the organism.

It is believed that under stress conditions, p53 primarily activates pro-survival mechanisms such as cell cycle arrest and autophagy. If DNA repair or restoration of homeostasis fails, pro-lethal processes are activated [12]. As a bidirectional regulator of cell death, p53 activates or inhibits its targets through transcription-dependent and transcription-independent mechanisms. Thus, p53 either induces or suppresses cell death, depending on a variety of factors.

### 2.1. Apoptosis

Apoptosis is currently the most studied type of cell death [13]. Interest in apoptosis is primarily because it does not cause inflammatory reactions, ensuring minimal damage to healthy tissues surrounding the dying cell, unlike other types of cell death [9].

**Figure 3:**
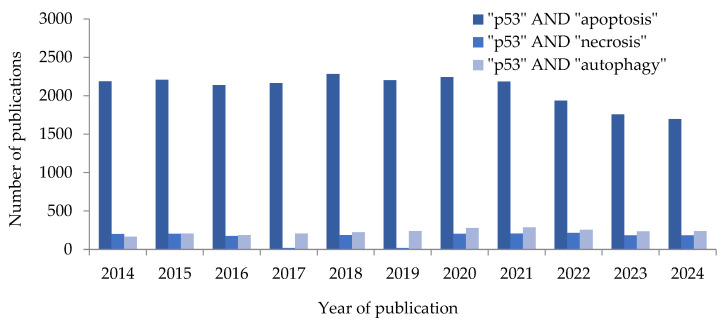
The number of scientific publications on requests in PubMed database. p53—transcription factor.

**Figure 4:**
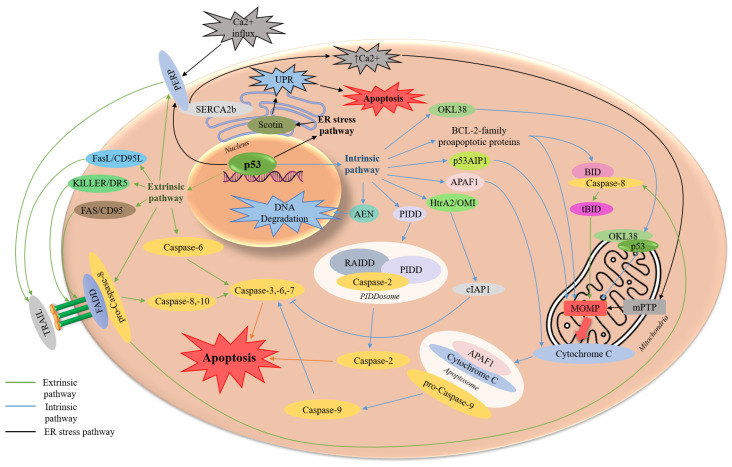
The transcriptional role of p53 in the regulation of apoptosis. AEN—apoptosis enhancing nuclease; APAF1—apoptotic peptidase activating factor; BCL-2—BCL2 apoptosis regulator; BID—BH3 interacting domain death agonist; tBID—truncated BID; cIAP—cellular inhibitor of apoptosis protein; ER—endoplasmic reticulum; FADD—Fas associated via death domain; FAS/CD95—Fas cell surface death receptor; FasL/CD95L—Fas ligand; HtrA2/OMI—HtrA serine peptidase; KILLER/DR5—DNA damage-inducible p53-regulated death receptor; MOMP—mitochondrial outer membrane permeabilization; mPTP—mitochondrial permeability transition pore; OKL38—oxidative stress induced growth inhibitor; p53—transcription factor; p53AIP1—p53 regulated apoptosis inducing protein; PERP—p53 apoptosis effector related to PMP22; PIDD—p53-induced death domain protein; RAIDD—RIP-associated ICH-1/CED-3-homologous protein with a death domain; SERCA2b—sarco(endo)plasmic reticulum calcium ATPase; Scotin—ER transmembrane pro-apoptotic protein; TRAIL—tumor necrosis factor-related apoptosis-inducing ligand; UPR—unfolded protein response. Straight arrow: activation; blunt arrow: inhibition.

In the context of anti-cancer therapy, p53 is considered mainly as an activator of apoptotic cell death. This is well illustrated by the high number of scientific publications on the topic in the PubMed database over the past 10 years (Figure 3) [14]. p53-dependent induction of apoptosis can occur via two partially interrelated pathways: extrinsic and intrinsic. A number of publications also describe the pathway of apoptosis caused by endoplasmic reticulum (ER) stress (Figure 4) [15,16].

The intrinsic (mitochondrial) pathway of apoptosis is characterized by mitochondrial outer membrane permeabilization (MOMP) and the formation of pores, leading to the release of hemoprotein cytochrome C [17,18]. Then, the apoptosome, formed from cytochrome C, APAF1 adapter, and initiator pro-caspase-9, activates effector caspases, further triggering the mechanisms of apoptotic cell death [19].

Numerous of p53 target genes and protein have been described within the intrinsic pathway. This is because p53 can regulate apoptosis via a transcription-dependent pathway in the cell nucleus, as well as a transcription-independent pathway by directly binding to protein targets on the mitochondrial outer membrane and in the cytosol. The transcriptional targets of p53 that are crucial for intrinsic apoptosis include BCL-2 family genes (*BIK*, *BCL2L11* (BIM), *BMF*, *BBC3* (PUMA), *PMAIP1* (NOXA), *BID*, *BAD*, *BNIP3*, *BECN1* (BECLIN1), *BAX*, *BAK1*, *BOK*), *TP53AIP* (p53AIP1), *APAF1* and *AEN*/*ISG20L1* [17,19,20,21].

The extrinsic, or receptor-dependent, apoptosis pathway, in turn, is initiated when extracellular death ligands bind to specific transmembrane death receptors (DRs) on the cell surface [18]. The subsequent binding of adapter proteins (e.g., FADD, TRADD, TRAF) leads to autoactivation of initiatory pro-caspase-8. Caspase-8 then activates downstream effector caspase-3 and caspase-7 to trigger a cascade of pro-apoptotic reactions [15,22].

To regulate the extrinsic pathway of apoptosis, p53 initiates transcription of DR genes such as *FAS* (CD95/APO-1) and *TNFRSF10B* (KILLER/DR5), as well as the ligand gene for the CD95/APO-1 receptor, *FasLG* (FASL/CD95L/APO-1L). *CASP6* (caspase-6) and *CASP8* (caspase-8) are also transcriptional targets of p53 [19].

In addition to the intrinsic and extrinsic pathways of regulating apoptosis, some researchers identify stress-related ER apoptosis that occurs as a result of the accumulation of misfolded proteins [23]. To eliminate the protein overload and restore homeostasis, cells activate specific unfolded protein response (UPR), which involves chaperones (e.g., BIP), transmembrane sensors (e.g., PERK, IRE1), and transcription factors (e.g., ATF4/6, CHOP) [23,24].

Under stress conditions, the regulatory domains of sensor proteins are located in the ER lumen, where they are bound to chaperones. As the ER load increases, the number of free chaperones in the lumen decreases, leading to the release of sensor proteins, their subsequent activation, and the initiation of the UPR [24]. If the UPR mechanisms fail to restore homeostasis, apoptosis is induced.

It is known that under ER stress, p53 suppresses *HSPA5* (BIP) translation. This reduces the ability of BIP to sequester pro-apoptotic BIK, leading to the subsequent induction of apoptosis [25]. It is also known that p53-mediated transcription of pro-apoptotic *SHISA5* (Scotin) induces apoptosis via a caspase-dependent pathway and suppresses the induction of autophagy, as Scotin is localized in the ER and modulates the UPR [26,27]. Another transcriptional target of p53 is *HtrA2*/*Omi*. In response to DNA damage, the mitochondrial serine protease HtrA2/Omi cleaves the anti-apoptotic cIAP1, thereby activating caspases and promoting apoptosis [28,29].

A further interesting pro-apoptotic target of p53 is *PERP*, a gene that encodes a transmembrane protein that acts as a mediator in the early stages of apoptosis, including the activation of the TRAIL-mediated extrinsic pathway of apoptosis [30]. It has also been shown that PERP accumulates Ca^2+^ in the ER by directly interacting with SERCA2b, promoting apoptosis. In addition, PERP can probably indirectly influence the activity of pro-apoptotic BID by activating upstream caspase-8 [31]. Another target of p53, *PIDD1*, plays its pro-apoptotic role by forming complexes with other proteins through its death domain. The PIDD1 adapter protein forms multi-protein complex called PIDDosome, also known as the caspase-2-PIDDosome. The PIDDosome, composed of PIDD1, RAIDD adapter, and caspase-2, activates pro-apoptotic caspase-2, which in turn promotes either apoptosis or cell cycle arrest in response to cellular stress [32,33].

In response to oxidative stress or DNA damage, p53 induces the transcription of *OSGIN1* (OKL38), which has both pro-apoptotic and antioxidant properties. In vitro, overexpressed OKL38 localizes to the mitochondria and interacts with mitochondrial p53, thereby promoting the release of cytochrome C [34]. Another target of p53 whose transcription is activated in response to DNA damage is *AEN*/*ISG20L1*, which encodes a nuclear pro-apoptotic nuclease [20,29]. The intrinsic pro-apoptotic mechanism of AEN/ISG20L1 involves DNA fragmentation [20].

In the context of anti-cancer therapy, the key disadvantage of apoptosis can be considered its reversibility even in the late stages (anastasis) [18]. The risk of reversibility of individual apoptotic events is a serious obstacle in the creation of drugs designed to induce apoptosis. Moreover, anastasis can be a contributing mechanism to the development of multidrug resistance [35,36].

### 2.2. Necrosis

Unlike apoptosis, necrosis is a pathological process that is closely related to inflammatory processes [15,37]. Necrosis, which may not involve caspase activation, is characterized by an increase in cell volume, swelling of organelles, membrane damage, and the subsequent release of intracellular contents into the intercellular space [15,38]. The rupture of plasma membrane and the release of intracellular components into the intercellular space cause an immune reaction and trigger inflammatory processes. Compared to other forms of cell death, necrosis poses the greatest threat to the patient’s life [4,39].

Necrosis includes such regulated forms of cell death as necroptosis, pyroptosis, and ferroptosis. The mechanisms of necroptosis are closely related to apoptosis, and upon inhibition of caspase-8 activity, the signaling pathway switches from apoptosis to necroptosis. Similarly to apoptosis, necroptosis can be induced both by Toll-like receptors (TLRs) and DRs (e.g., TNFR1). Pyroptosis, an inflammatory caspase-dependent process, can be induced by inflammasomes (e.g., NLRP3), which can also induce apoptosis in certain cases. In contrast, ferroptosis is an ancient mechanism of specific lipid peroxidation, which leads to the rupture of the plasma membrane and the so-called synchronized regulated necrosis (SRN) of cells [39].

The most studied form of programmed necrosis is necroptosis. Necroptosis is accompanied by the RIPK1-mediated activation of the RIPK3 serine-threonine kinase. This kinase then phosphorylates the MLKL pseudokinase, which leads to membrane permeabilization [37,38,40]). Necroptosis occurs when apoptosis is suppressed, often by inhibiting the activity of caspase-8, which prevents it from cleaving RIPK1 and RIPK3 [38,40]. Necroptosis can be initiated by the same death ligands that activate the extrinsic pathway of apoptosis: TNF-α, FasL, TRAIL, and TLR3/4 [40].

Pyroptosis, in turn, is characterized by the formation of plasma membrane pores, followed by cell swelling, rupture of plasma membrane, and the release of cytoplasmic proteins such as lactate dehydrogenase (LDH), damage-associated molecular patterns (DAMPs), and pro-inflammatory cytokines interleukin-1β (IL-1β) and interleukin-18 (IL-18) [41]. Pyroptosis is mediated by multiprotein complexes, inflammasomes, which consist of a pro-caspase (usually pro-caspase-1), an adapter protein (e.g., ASC), and a sensory protein (a member of the NLR or AIM2 family). Pyroptosis involves the caspase-dependent cleavage of gasdermin proteins (GSDMA, GSDMB, GSDMC, GSDMD, GSDME, and GSDMF) into N-terminal and C-terminal fragments. Notably, the inflammatory caspase-1, -4 and -5 cleaves GSDMD, while the pro-apoptotic caspase-3 cleaves GSDME [42,43]. The N-terminal fragments of GSDMD (N-GSDMD) and GSDME (N-GSDME), released by cleavage, form pores in the plasma membrane, leading to necrotic cell death [39,44,45].

Ferroptosis is a form of cell death that is independent of caspase activity and is induced by the oxidation of Fe^2+^ to Fe^3+^ as a result of the Fenton reaction. Ferroptosis is mediated by the accumulation of reactive oxygen species (ROS) that results from a decrease in GPX4 activity and intracellular glutathione (GSH) levels [46,47]. The accumulation of reactive oxygen species (ROS) triggers a chain lipid peroxidation reaction of polyunsaturated fatty acids (PUFAs) in cell membranes, resulting in membrane disruption, increased permeability, and ultimately, cell death [47,48]. A non-canonical GPX4-independent ferroptosis pathway has also been identified [48]. Interestingly, cells that have escaped other types of cell death may become hypersensitive to ferroptosis. That is why its therapeutic induction in anti-cancer therapy has attracted a lot of attention [46].

**Figure 5:**
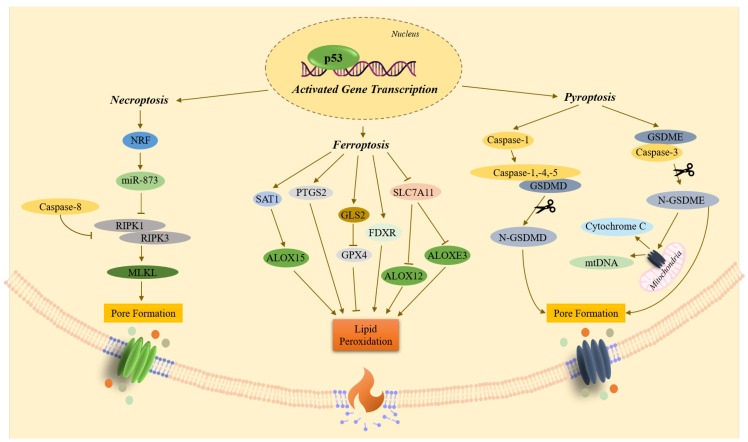
The transcriptional role of p53 in the regulation of necrosis. ALOXE3,-12,-15—arachidonate lipoxygenases; FDXR—ferredoxin reductase; GLS2—glutaminase; GPX4—glutathione peroxidase; GSDMD,-E—gasdermins; N-GSDMD,-E—N-terminal fragments of gasdermins; miR-873—microRNA, post-transcriptional regulator; MLKL—mixed lineage kinase domain-like pseudokinase; mtDNA—mitochondrial DNA; NRF—transcriptional repressor; p53—transcription factor; PTGS2—prostaglandin-endoperoxide synthase; RIPK1,-3—receptor interacting serine/threonine kinases; SAT1—spermidine/spermine N1-acetyltransferase; SLC7A11—membrane transport protein. Straight arrow: activation; blunt arrow: inhibition. Scissors represent the cleavage of a protein.

The role of p53 in necrosis is not described in such detail as in apoptosis (Figure 5). As noted above, p53 positively regulates pro-apoptotic BAX/BID proteins at the transcriptional level or binds to them directly, contributing to the formation of pores and MOMP. This process can also lead to mitochondrial necrosis [11,49]. p53 also transactivates *NKRF* (NRF). The transcriptional repressor NRF suppresses microRNA-873 (miR-873), thereby leading to an increase in RIPK1 and RIPK3 levels and triggering necroptosis [16].

The transcriptional function of p53 in pyroptosis consists primarily in the induction of *GSDME*. The cleaved form of this pore-forming protein can also form pores both in the mitochondrial membrane, promoting the release of pro-apoptotic cytochrome C and mitochondrial DNA (mtDNA), and in the plasma membrane [41]. p53 also transcriptionally activates *CASP1* (caspase-1). Active caspase-1 then cleaves the pore-forming protein GSDMD, leading the resulting N-GSDMD fragment to form pores in the plasma membrane as well [16,41,44,50].

Under oxidative stress or doxorubicin treatment, p53 promotes ferroptosis by transcriptionally regulating the p53/*SAT1*/ALOX15 pathway [48,51,52]. Furthermore, under the action of synthetic ferroptosis inductors RSL3 or Erastin, p53 transactivates *FDXR* to promote ferroptosis [48]. p53 also suppresses the transcription of *SLC7A11*, thereby increasing cell sensitivity to ferroptosis via GPX4-independent pathways p53/*SLC7A11*/ALOX12 and p53/*SLC7A11*/ALOXE3 [48,52]. Another p53-dependent mechanism of positive regulation of ferroptosis is transactivation of glutaminase-2 (*GLS2*) that catalyzes the deamination of glutamine to glutamate. Glutamate, along with glycine and cysteine, is a key component of glutathione (GSH). When *GLS2* is transactivated, it can lead to low levels of GSH, which in turn inhibit the activity of GPX4, the key inhibitor of ferroptosis [46,48]. In addition, p53 promotes ferroptosis by transactivating *PTGS2* (COX2) [48].

### 2.3. Autophagy

In cancer, where apoptosis mechanisms are disrupted, autophagy can also be inhibited [38]. In eukaryotic cells, autophagy is the main pathway of lysosomal degradation of long-lived proteins and cytoplasmic organelles. Autophagy primarily serves as a cell survival mechanism in response to nutrient deprivation, hypoxia, and ER stress. In this context, autophagy frequently suppresses apoptosis and necrosis, but under specific conditions it can also promote cell death [4,15]. Furthermore, the anti-necrotic function of autophagy plays an important biological role in various diseases, including cancer [15].

Autophagy is characterized by the formation of multiple autophagosomes, as well as a decrease in mitochondrial size and ER area. Autophagy is performed by isolating and sequestering cytoplasmic components within two-membrane structures called autophagosomes. Autophagosomes then fuse with lysosomes to form autolysosomes, where the contents are cleaved by hydrolases [4,53].

Autophagy can also cause cell death, as the fusion of autophagosomes with lysosomes can lead to the destruction of almost all membrane organelles. Autophagy-dependent cell death is characterized by several features, including partial chromatin condensation and sometimes pyknosis of the nucleus. It is also distinguished by a lack of nuclear and cellular fragmentation in the late stages of death, as well as a lack of DNA degradation to the nucleosome level. Other characteristics are an increased number of autophagosomes and autolysosomes, higher lysosomal activity, and an elongated Golgi apparatus. Sometimes there is an expansion of the ER cisterns, an increase in mitochondrial permeability, and prolonged preservation of microtubules and intermediate filaments, and the inactivity of caspases. Following autophagic cell death, the cellular debris is cleared by macrophages [54].

Similarly to its role in apoptosis and necrosis, p53 regulates both the promotion and inhibition of autophagy, depending on its subcellular localization [16]. Thus, nuclear p53 stimulates autophagy, whereas cytoplasmic p53 suppresses it [16,55].

**Figure 6:**
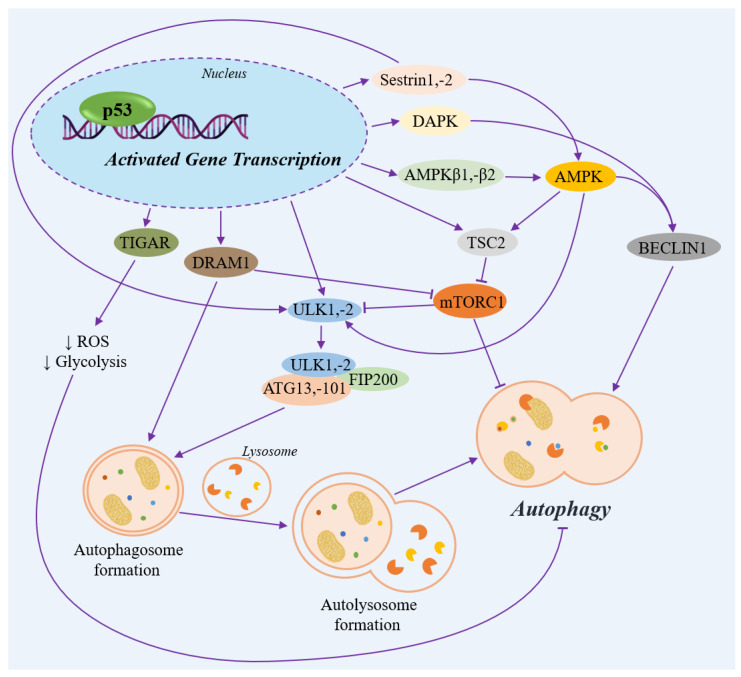
The transcription-mediated p53 signaling pathway of autophagy. AMPK—AMP-activated protein kinase; ATG13,-101—autophagy related proteins; BECLIN1—autophagy regulator; DAPK—death associated protein kinase; DRAM1—damage regulated autophagy modulator; FIP200—FAK family interacting protein; mTOR1—mechanistic target of rapamycin kinase; p53—transcription factor; ROS—reactive oxygen species; Sestrin1,-2—regulators of cellular metabolism and adaptation to stress; TIGAR—TP53 induced glycolysis regulatory phosphatase; TSC2—TSC complex subunit; ULK1,-2—Unc-51 like autophagy activating kinases. Straight purple arrow: activation; blunt purple arrow: inhibition; downward black arrow: inhibition of a process.

Under non-stressed conditions, p53 inhibits autophagic cell death through the indirect negative regulation of BECLIN1, an adapter protein essential for the formation and maturation of autophagosomes. p53 also transcriptionally activates *TIGAR*. TIGAR bisphosphatase, in turn, suppresses the formation of ROS and glycolysis, thereby inhibiting the process of autophagy (Figure 6) [12].

In response to genotoxic stress, p53 promotes autophagy by transactivating *SESN1* (Sestrin1) and *SESN2* (Sestrin2) [12,56]. Sestrin1 and Sestrin2 bind directly to AMPK, the central regulator of energy homeostasis [57], promoting the AMPK-mediated activation of the TSC2 complex. This, in turn, ultimately suppresses mTORC1, a negative regulator of autophagy [12]. Sestrin1 and Sestrin2 also bind to ULK1, the serine/threonine kinase that serves as a key initiator of autophagy [58].

Besides its indirect role, p53 can also transcriptionally activate the AMPK subunits β1 and β2 (*PRKAB1* and *PRKAB2*), as well as *TSC2* [12]. In turn, AMPK promotes the phosphorylation of BECLIN1 at S90 and S93, a modification that leads to autophagy in response to glucose deficiency [59].

Autophagy initiation can also occur via the p53-dependent transactivation of *DAPK1* and *DRAM1*. DAPK1 activates the autophagy protein BECLIN1, prompting its release from Bcl-XL-mediated sequestration [12]. DRAM1 is a lysosomal protein that appears to be involved in the formation of autophagosomes and inhibits the mTOR pathway, leading to autophagy [55,60]. DAPK1 and DRAM1 are both involved in the mechanisms of apoptotic cell death [12].

The ULK family of serine/threonine kinases (*ULK1* and *ULK2*) is best known for its regulatory role in the initiation of autophagy, which can be transcriptionally mediated by p53 [56,61,62]. ULK1/2 binds to autophagy-related (ATG) proteins to form the complex, which includes ULK1/2, FIP200, ATG13/101. Further, the ULK–FIP200–ATG triad complex participates in the formation of autophagosomes [62,63,64].

It is assumed that in the late stages of cancer development, autophagy can suppress p53 activity. This contributes to oncogenesis, given that p53 deficiency induces a form of autophagy that does not lead to cell death [4,56,65]. Within anti-cancer therapy, autophagy inhibitors play an important role because they increase the sensitivity of cells to treatment and promote apoptosis [4].

## 3. p53 Regulates the Relationship Between Apoptosis, Necrosis, and Autophagy

As a key regulator of apoptosis, necrosis, and autophagy, p53 balances cell survival and cell death in response to various stress factors. Although necrosis and apoptosis are known to involve similar biochemical processes, they are usually not considered as interrelated mechanisms in most cell death studies [4,13]. Due to interconnected downstream signaling pathways, both apoptosis and necrosis can activate caspase and non-caspase proteases [4,9,15].

For example, the protein DRP1, which is encoded by *DNM1L*, can be involved in both apoptosis and necrosis depending on the specific stress and cellular context. Thus, nuclear p53 transactivates *DNM1L* (DRP1), which controls the permeability of MOMP and promotes apoptosis. Conversely, cytoplasmic p53 forms a complex with DRP1, causing its translocation into mitochondria and the subsequent opening of the mPTP and necrosis [37,66]. Evidence also suggests that DRP1 is involved in mitophagy in cardiomyocytes, where it acts like a BH3-only protein and disrupts the interaction between BECLIN1, a regulator of autophagy, and Bcl-XL, an anti-apoptotic protein [66].

Another ambiguous target transcriptionally regulated by p53 is *CTSD* (cathepsin D). The lysosomal protease cathepsin D, in turn, regulates apoptosis by triggering BAX insertion into the mitochondrial membrane through BID cleavage, which causes cytochrome C release and caspase-3 activation. However, cathepsin D can also indirectly shift the apoptotic process towards necroptosis [67].

Caspase-3 and caspase-8, in turn, can play a key role in inducing both apoptosis and pyroptosis [44,68]. Pro-inflammatory caspase-1 is capable of inducing apoptosis in the absence of GSDMD. There is also a relationship between pyroptosis and necroptosis, as MLKL can activate the NLRP3, promoting the maturation of pro-inflammatory cytokines IL-1β and IL-18 [44].

A hypothesis by D. Lemasters (1999) describes necroptosis as a link between the processes of apoptosis and necrosis [9]. This hypothesis is supported by the observation that the mPTP opens in both necroptosis and apoptosis. In this case, the choice of cell death pathway depends on the cellular ATP level. Thus, if mitochondrial dysfunction leads to significant depletion of ATP stores, necrotic cell death occurs. Conversely, if ATP levels increase, apoptosis occurs [69].

It is also known that the pro-apoptotic BH3-only proteins NOXA, BAX, BAK1, BNIP3 and BAD can cause autophagy by releasing BECLIN1 from the complex with anti-apoptotic BCL-2/Bcl-XL [4,12,16,70]. Pro-apoptotic PUMA can induce mitophagy, a process that occurs simultaneously with the release of cytochrome C [70]. In addition, it has been shown that the apoptotic nuclease AEN/ISG20L1 is involved in autophagy under genotoxic stress [71,72]. The transcriptional targets of p53 associated with both apoptosis and autophagy also include *p14ARF* and *TP53AIP1* (p53AIP1) [73,74]. Furthermore, a reverse mechanism exists where caspase-3 and caspase-8 can cleave BECLIN1, thereby shifting the process from autophagy toward apoptosis [4].

The connection between autophagy and necrosis, in turn, has not been definitively proven but also has not been ruled out. It is assumed that, under genotoxic stress and ATP depletion, PARP1 induces necrosis, whereas AMPK inhibits the mTORC1 pathway to promote autophagy [4].

Beyond the transcription-dependent pathways discussed above, this review does not cover other important factors that influence p53-dependent cell death pathway choice, such as protein–protein interactions involving p53, post-translational modifications of p53, isoforms of p53, and long non-coding RNAs (lncRNAs) associated with p53 transcriptional activity [3,75]. Such a variety of factors makes the study and classification of cell death processes a complex task. Ultimately, the mechanism of cell death depends on the cell type, the cellular microenvironment, and the initial inducers [4].

## 4. Markers for p53-Mediated Cell Death

Although p53 activation is an attractive anti-cancer strategy, it is necessary to assess the risks of its various consequences. p53-dependent autophagy or cell cycle arrest can subsequently promote tumor recurrence and the development of secondary chemoresistance. Necrosis, in turn, is associated with inflammatory reactions in the surrounding tissues.

Although classical methods can confirm programmed cell death, they do not reliably distinguish between its different types. MTT, XTT, RSB and similar colorimetric methods can only detect a decrease in cellular metabolic activity [76,77,78]. While Annexin V and 7-AAD can detect membrane permeability changes, they do not specify the pathway of cell death [79].

Although p53 is directly or indirectly involved in the activation of main types of cell death, distinct key markers have been identified for each type. These markers can be used to develop anti-cancer compounds (Table 1).

There are a number of methods for identifying a specific type of cell death, primarily apoptosis [80]. However, complementary methods are usually required, particularly the analysis of inherent morphological changes in cells using microscopy. For example, apoptotic cell death can be confirmed by demonstrating DNA fragmentation and the activation of relevant caspases. The most informative analysis of caspase activation, in turn, involves detecting the cleaved products of their target molecules, such as the cleaved form of PARP1 (before cleavage—116 kDa, after—85 and 31 kDa) or Lamin A/C (before cleavage—74 kDa, after—46 and 28 kDa) [81]. However, as the *CASP3* and *CASP7* are mutated in some tumors, their analysis may be complicated [82].

In published studies, the analysis of p53-related pro-apoptotic processes frequently focuses on the cleavage of caspase-3, PARP1, cytokeratin-18 (CK18-M30), and Lamin A/C, as well as a decrease in the expression levels of apoptosis inhibitors (IAPs) such as *BIRC5* (Survivin) и *XIAP* [81,82,83,84,85].

Despite morphological differences, apoptosis and necrosis share key mechanisms, suggesting that no clear biochemical distinction can be made between them [86]. Although phosphorylation of RIPK1 and RIPK3 and their downstream target MLKL serves as a marker for necroptosis in several scientific studies, RIPK3 and MLKL can also regulate both inflammasomes and pyroptosis [39].

Markers of pyroptosis include the activated caspase-1, -3, -4 and -5, as well as the cleaved fragments of GSDMD and GSDME [39,45]. Ferroptosis markers usually include the presence of lipid peroxidation enzymes from the ALOX family (ALOX12, ALOX15, ALOXE3), a reduction in *SLC7A11* mRNA, and a decrease in GPX4 activity [51,52]. Analysis of *PTGS2* (COX2) mRNA expression is used as a pharmacodynamic marker, but it is not a strictly selective marker of ferroptosis [51].

Autophagy-dependent cell death is characterized by extensive vacuolization of cytoplasm and accumulation of autophagosomes. This process can be detected via the phosphorylation status of ULK proteins and quantified using the LDH sequestration assay to measure autophagic flux [87].

To confirm a particular cell death modality, the use of specific inhibitors of the processes under study is recommended. For example, Q-VD-Oph serves as a potent broad-spectrum caspase inhibitor for apoptosis. Necrostatin-1 (Nec-1) suppresses necroptosis via RIPK1 inhibition, whereas ferrostatin-1 (Fer-1) effectively inhibits iron-dependent ferroptosis. Pyroptosis can be blocked by disulfiram (DSF), which selectively targets GSDMD. To inhibit autophagy, chloroquine (CQ) is applied to arrest autophagic flux [85,88].

**Table 1:** Marker combinations for distinguishing between p53-mediated cell death modalities.

Type of Cell Death		Distinctive Morphological Feature	Primary Biochemical Marker	Selective Chemical Inhibitor	Reference
Apoptosis		Membrane blebbing, formation of apoptotic bodies, cell shrinkage, nuclear fragmentation, chromatin condensation	- cleaved fragments of caspase-3, - cleaved fragments of PARP1, - cleaved fragments of CK18-M30, - cleaved fragments of Lamin A/C - *↓ BIRC5* - *↓ XIAP*	Q-VD-Oph	[81,82,83,84,85,89]
Necrosis	Necroptosis	Increased membrane permeability, cell swelling, plasma membrane rupture, pore formation	- inhibited caspase-8 - activated RIPK1, RIPK3 - activated MLKL	Necrostatin-1	[39,88,89]
	Pyroptosis	Cell swelling, plasma membrane rupture, large pore formation	- activated caspase-1, -3, -4 and -5 - cleaved fragments of GSDMD, GSDME	Disulfiram	[39,45,88,89]
	Ferroptosis	Plasma membrane rupture	- *↓ SLC7A11* - *↑ PTGS2* - *↑* ALOX15, ALOX12, ALOXE3 - *↓* GPX4	Ferrostatin-1	[51,52,88,89]
Autophagy		A decrease in the number of mitochondria and the volume of ER, a change in the size of the Golgi apparatus, the formation of autophagosomes and autolysosomes	- activated ULK - sequestration of LDH	Chloroquine	[85,87,89]

↑—increased mRNA or protein level; ↓—decreased mRNA or protein level.

## 5. Conclusions and Perspectives

Forty years after its identification as a tumor suppressor, p53 remains a highly attractive target for anti-cancer therapy. As a transcription factor, p53 regulates numerous genes, including those involved in the initiation and progression of cell death. However, a major challenge for reactivating the transcriptional activity of p53 with small molecules is twofold: the difficulty of inducing a specific cell death pathway and the inability to accurately determine its type using common methods of molecular and cellular biology.

This article reviews the most frequently investigated transcription targets of p53 involved in apoptosis, necrosis, and autophagy, along with common genes and markers for these three types of cell death. The presented information highlights the importance of identifying the specific p53-mediated mechanism during preclinical studies of new p53 reactivator candidates. This understanding is essential for the rational design of selective therapeutic compounds that induce cancer cell death while mitigating potential adverse effects.

## Data Availability

No new data were created or analyzed in this study.
